# Filling of Canals and Pulp Cavities

**Published:** 1897-01

**Authors:** J. Foster Flagg

**Affiliations:** Philadelphia.


AMERICAN JOURNAL

—OF—

DENTAL SCIENCE.

VOL. XXX. BALTIMORE, JANUARY, 1897. NO. 9.

ARTICLE I.

### FILLING OF CANALS AND PULP CAVITIES.

BY J. FOSTER FLAGG, D. D. S., PHILADELPHIA.

In concluding the paper on the "Preparation of Pulp Cavities and Canals," which I read before you at your last year's meeting, I said the work which dentistry has to do, after pulp cavities and canals are prepared, eventuates in such varied and diverse practice as would require for its discussion a paper longer than the one then presented. But what I desired to leave with you then for your thoughtful consideration was :

First. That other things besides the thorough extirpation of pulp tissue are of infinitely more importance in connection with canal work.

Now, I ask you, is it not the universal teaching that you are to thoroughly extirpate the pulp first and foremost? You are to reach the end of every canal if possible, and you are to thoroughly extirpate that pulp. That is what has been held up to you as the result, *par excellence*, to attain which you are to concentrate all your energy ?

Second. "That in the effort to accomplish this *comparatively* unimportant result"—

Now, why do I call this a comparatively unimportant result? You may remember that I said that it would be better that the pulp tissue should not be touched, better that you should leave it entirely in the pulp cavity, and put in your filling knowing that you had not touched the de-devitalized pulp, *than* that you should do certain other things which you might do; and therefore it is that this extirpation of the pulp tissue is *comparatively* unimportant.

Second. That in the effort to accomplish this *comparatively* unimportant result, frequently irreparable injuries are inflicted.

And this is the idea which I wish to leave with you solid this year.

Third. That the idea that any work, no matter how thoroughly or perfectly done *inside the tooth*, will preclude the possibility of future trouble is fallacious, and for the benefit of semi-vital teeth should so be taught.

Fourth. That it should be recognized that all the other numerous causes of periodontitis, besides putrescence of the pulp, are more likely to produce that trouble with semi-vital than with wholly-vital teeth.

You know there are sixteen or seventeen causes of periodontitis; putrescence of the pulp is *only one* cause.

Fifth. That in recognition of all this, pulp cavity and canal work should be such as would permit of future reparation if there be deficiencies, and of prompt and easy relief in the event of future periodontitis.

So far from such views being generally accepted, it seems to me that the whole line of thought, teachings and work, in this connection, as understood and practiced is diametrically antagonistic to them, based upon no foundation, so far as indications are concerned, and productive of no other results than those of liklihood of irremediable detriment and oftentimes of positive prevention of long-continued tooth conservation; and all this is *taught* from almost every chair of operative dentistry as the "*best!*"

I was fortunate in escaping from this thraldom thirty years ago, but I could not bring you the experience and the *results* of all these years had I not waited for them!

I feel that it was owing to an entire change of practice, together with the line of thought which inaugurated and sustained methods utterly at variance with all former, and largely of present ideas, that I have been able to establish what reputation I have as a treater of teeth, and that I have been able to impress the truths of my lectures, in this connection, by clinical examples of long-continued maintenance for usefulness of teeth which had been abandoned as "hopeless" by those who had achieved the title of "eminent" in our profession.

It is this which impels me to bring before you the paper which I now present for your consideration, Having entered and prepared the pulp cavity and one or more canals pertaining to any given tooth, the next step is one which certainly has given dentistry a theme for voluminous discussion.

That these canals shall be filled seems to be practically universally conceded, and yet it has been contended by some that filling is not necessary, and experiments with *fully formed* teeth show unfilled canals free from moisture, even though the teeth had been immersed in clear water (much thinner than serum) for many months, and yet that the canals shall be filled seems to be conceded, and for myself I should not think of letting a canal go unfilled if I could fill it.

But *for what* is it to be filled? That is the question which I ask you. Upon this question dentistry is divided; were it not so we should not read that some fill with gold; some with amalgam; some with oxychloride; some with chloro-percha; some with wire; some with hickory sticks; some with salol; some with balsamo del deserto; some with cotton, and some with medicated pastes. This variance rivals the shotgun prescriptions of genenal medicine and should make dentistry ashamed!

I have said "*for* what" and not *with* what, advisedly, for in dentistry as much, if not more, possibly, than in almost any other calling, the means utilized are with an accurate view to the accomplishment of some specific end.

Now, if gold be indicated, cotton can hardly be favorably regarded; if chloro-percha be the desirable thing a hickory stick will make but a doubtful substitute, so the question is very pertinent: for what is the canal and pulp cavity filled?

In dental work it will, I think, be conceded that the effort is ever to replace that which is lost with something which will, as far as is practicable, supply the deficiency, and, if possible, make things better than they were before.

It, therefore, behooves us to consider the changes incident to the loss of the pulp, that we may have a definite idea as to what it is which we are to try to replace.

What are the functions of the pulp?

First. The formation of dentine and the maintenance of its validity.

Second. The induction of "tubular consolidation" as protection against pulp irritation concomitant with advancing caries.

Third. The recalcification of decalcified dentine.

Now think what a work this is? Only think how the teachings have changed since the old days when Professor Arthur was tabooed and ever-to-be-sat-down-upon, because of the fact that he advocated leaving decalcified dentine in cavities and filling over such decalcifications! If a pulp was to be endangered by the removal of decalcified dentine, then the decalcified dentine was not to be removed because the pulp would recalcify it. What is the advance teaching of to-day? Is it that the less decalcified dentine there be removed the better; it is that the only removal of decalcified dentine is of such as will militate against the integrity of the filling. The pulp is not thought of, no one says a word about it; we only recognize that you leave a large mass of decalcified dentine within the cavity and fill over it. So that the integrity of the filling is not interfered with, the dentine will be recalcified and the tooth in six or eight years, will be in a better condition than it was before.

Fourth. The formation of secondary dentine, when, from attrition, deep-seated caries or fracture, such loss of dentine has occurred as necessitates for pulp conservation such deposition within the physiological pulp cavity.

It is almost superfluous for me to say that with the loss of the pulp we lose every one of its attributes, and that we are unable, in any degree, to supply its place.

Now, this is a serious proposition. When we lose the pulp of a tooth we lose an organ, the attributes of which we can in no wise replace. We can do nothing which shall accomplish one thing which the pulp did.

Of all the efforts on the part of dentistry this is unquestionably, the least successful, but, if we cannot do as well as we could wish, and as well as we do in every other direction, what yet remains for us to consider, and how can we best make some amend for the great loss which the tooth has sustained? That highly organized tissue, the dentine, has lost its vitality; the root portion of this tissue rests against living cementum nourished by pericemental membrane; this dentine, being dead, must gradually become changed from an aseptic to a septic substance, which necessarily must, in time, become an irritant to the cementum and its membrane.

The preservation of this dentine is then *the only thing* that we can *try* to do in place of all that could be done by the pulp which has been lost.

But again, there is a possible influx of moisture, under circumstances entirely different from those which formerly existed. Naturally the pulp cavity was always full of moisture, but it was the moisture of nutrition, the moisture which came and went, and which was constantly renewed in its subservience to life work.

Now all is different. What moisture should find its way into the empty canals and pulp cavity wound find no egress, and would but add more material for decomposition and to evolution of mephitic gas. Hence the prevention of ingress of moisture would seem, ordinarily, to be another reason for filling canals and pulp cavities.

Are there any other reasons? If so, I would ask for them, for no others have ever presented themselves to me.

And now it is in place to discuss upon some tangible basis the merits of the various materials used for this purpose. First, let us consider gold as the one which has been longest used and the one which even to-day is largely taught and regarded as the "best."

Why is it the best? What single attribute has it which makes it good at all?

That is certainly a very pertinent question!

Can it do anything toward preserving the integrity of the dead dentine? Is it any better than tinfoil or facing amalgam as a barrier against the ingress of moisture?

If it has no antiseptic value, why is it needful to fill the whole canal and entire pulp cavity with it? If exclusion of moisture is the object sought, how far does moisture creep beyond the first properly inserted and consolidated pieces? And if it does not get beyond them what is the object of all the rest?

Certainly many of us have filled canals as solidly as possible with gold. I have filled many canals as solidly as I could, thus making what I was then taught to be, and what I then believed to be, thorough operations of the very best kind, and it cannot be disputed that many men do that to-day.

It was partly because I could give myself no satisfactory answer to these questions that I early abandoned gold as a filling material for canals and pulp cavities, and that I have made no such use of it in thousands of teeth which I have since treated, and certainly no such use of it in the thousands of teeth which I have, with great labor and difficulty, re-treated in which it had proven unavailing, although introduced with marvelous manipulative ability.

Next, I relinquished amalgam and also dissolved base plate (now called chlora-percha), because with a few years of experience with them during the late '50's and early '60's I had come to regard them as the worst materials ever employed for this purpose, an opinion to which I adhere now, most decidedly, as a deduction from the nearly *forty years* of canal work, in which I have indulged as a practitioner, and demonstrated in my clinics as a teacher,

As with gold, neither of these materials possess one valuable attribute, and both possess the very objectionable ones of easy insinuation into very fine and inaccessible canals, and the impossibility of subsequent removal, should removal, for any reason, ever be necessary.

Properly pointed and nicely introduced hickory sticks and gold wires were the first deviations from the solid fillings of canals, and, though the idea of "filling space" was still adhered to in the bulbous portions, the recognition of the easy removal seemed to be the first knowledge of a requirement, which, by 1865 or 1870 had been forced upon the observation of some few dentists.

By this time my practice was almost exclusively confined to "treating teeth," and even this was again restricted to the poorest, softest and least properly calcified, as with these, *clinical experience* had amply shown the value of "choice of filling material" and that this choice was practically confined to the "plastics."

I have been asked to-day if I would state once more upon this floor, because some few, as yet, are not aware of the fact, that my reason for abandoning gold was because I abandoned the treatment of any such teeth as I judged needed gold filling.

Whenever a tooth came to me of such structure that I or any dentist, would fill it with gold, I was in the habit of saying to the patient, "I have too many teeth of the poorest, softest and most troublesome kind to take care of; I cannot spend my time taking care of such teeth as yours. They can be nicely treated, easily filled and would do you good service." So it was that I abandoned the use of gold; not because I did not know thoroughly, as I have stated to you, the advantages that gold has, and that it is the only one filling material upon which there is no discount whatever when it is properly used.

Thus it was, that being constantly brought in contact with non resistant, non-recuperative tissues, I had vastly more than usual opportunity for noting the results of varied pulp cavity and canal work, both in my own hands and from those of many excellent practitioners. Then it was that the notation of results had already begun to assume large proportions, and that I was able to *know* that of six thousand cases of reasonably and even excellently "well-treated teeth," nine in every ten gave trouble in less than twenty years.

Now, I want you, friends, to think over that statement. You know that I do not make statements unless I have the facts. I have followed six thousand cases of treated teeth which had to be re-treated, six thousand cases of teeth in connection with which pulp cavity or canal work had been from some cause or another a feature in the treatment, and in those six thousand cases, of which some were excellently well treated, others not so well, and others poorly treated, nine in every ten had given trouble before twenty years of that tooth's life had passed over.

In this line of work three-fifths of these peridental irritations had to be credited, most largely, to the *length of time during which the peridentium had been doing double duty* as a nourisher of tissues in contact with dead tissue—the dentine of pulpless teeth,

It is in this connection that I desire to impress the first reason for filling canals and pulp cavities, for it was noted that just in proportion as effort had been made to maintain the integrity of the dead dentine, so treatment was successful in the most unpromising cases.

It is now important that the very decided difference between "pulpless teeth" and teeth containing portions of remaining pulp should be clearly recognized, for, when it is remembered how very frequently teeth, in which pulp has been devitalized, are spoken of by those who performed these operations as "dead teeth," it should be accepted that cases of almost complete extirpation of the pulp might as frequently be referred to as "pulpless teeth."

Cases of peridental irritation from putrescent pulp are therefore *never* to be regarded as from pulpless teeth, but always from teeth containing a sufficient quantity (be it great or small) of very objectionable pulp.

This brings us to the statement that in a vast majority of peridental irritations associated with semi-visal teeth, more relief is given by opening canals than by any other means used, and *frequently* more relief is given by this means than all other means combined, and this no matter from what cause.

Indeed, so potent a factor in treatment is this opening of canals that in some instances teeth will remain comfortable only so long as canals remain open, and persistently give trouble almost immediately upon the stopping of the tooth.

You all readily recognize that in making this statement I bear in mind the great improvement which has occurred in the treatment of such teeth. Twenty-five or thirty years ago we frequently met with teeth which would not bear stopping up; ten years ago there were less, and to-day we meet with very few, but still we meet them. In such cases a "vent," either through the tooth or gum, must be established, or the more radical operation of extraction and replantation performed with the hope, and sometime with the result, of success.

But it must also be recognized that with every such infliction a greater amount of secondary or scar tissue supervenes, and that with this the liability to future trouble is increased proportionately.

Nearly twenty years ago (*Cosmos*, November, 1877) I wrote: "It has been decided that a pulpless tooth is far better than no tooth, but it is equally decided that sooner or later, subject to a variety of contingencies, trouble will *probably* develop, and that it behooves every practitioner to so act as will best provide against the occurrence of this probable evil.

The added nineteen years of experience has but thoroughly confirmed this opinion, and has made warnings to my classes only the more positive and the more emphatic.

This, in turn, brings us to the consideration of other than reasons for the *filling* of canals and pulp cavities, as it is seen that probable need of *unfilling* may exist; therefore, it is that I would give you as of importance:

First. That canals should be filled with medicaments, non-poisonous, non-irritating, soothing and of *long-continued* antiseptic power, and that these should be introduced in fluid, semi fluid or paste form, or, when possible, upon a vehicle which absolutely maintains its integrity.

Second. That the filling should *fill* as perfectly as possible in order that while maintaining anti-sepsis it should aim to preclude, as much as possible, the ingress of moisture.

Third. The filling should be easy of introduction, not that this is an essential, but that, everything else being equal, it is certainly desirable.

Fourth. That it should be easy of removal, which attribute, while it is of but little moment at the time of filling, becomes of *paramount importance to the patient*, if, in the course of time, it means the long continuance of suffering, or the affording of comparatively prompt relief.

Should our patients never be thought of? Should they not be thought of first, last and all the time? In thinking of them most emphatically we think of ourselves, in doing for them we do for ourselves.

The medicaments of which I can best speak are those which I have longest used. In my experience no others have been more satisfactory than the oil of cinnamon and the oil of cloves as antiseptics, and the acetate of morphia and sulphite of lime as paste makers. For some of the medicaments, which, at suggestion, I have used in canal work, such as creosote, oily carbolic acid, bichloride of mercury and salicylic acid I can only say that I have entirely discarded them, while for iodoform I have but scanty words of praise, and most infrequent use.

I have *no* use for bedbug poison in my office!

The modern medicaments, such as eugenol, camphophenique and fluo-silicate of soda (sodium silico-fluoride) have made excellent records during the last decade, but these must do many years of good work before they can be ranked as *better* than those that have already done from twenty to thirty years' service.

For the newest and most extensive line of germicidal antiseptics, with all their ingenuity of manufacture and compounding, with all their comparative tests of merit and their long lists of testimonials, we can but say that the same long and careful trials as have been given to the "old reliables" must be given to them before their value can be positively established, while it certainly cannot but be noticed that most of those who testify enthuastically are those who have no long recoid of continuous methods, but who are disposed constantly to favor new things.

Notwithstanding the occasional assertion of a few that they enter, extirpate from and fill to the extremity of even the finest and most tortuous canals, I think that it will not be disputed that we, of the large majority, do not do so.

There are three usual grades of canals; those which are accessible, large, easily entered, and which can be explored to their ends without difficulty; those that are yet accessible, but which are small, not easily entered and which it is always difficult, and sometimes impossible, to satisfactorily explore, and finally those which are much less accessible, almost or quite impossible to enter, and absolutely impossible to explore for any distance, either on account of fineness or tortousity.

Besides these there are yet other canals, which although neither inaccessible, nor small, are found in roots, the shapes of which preclude the possibility of following by either drill, broach or finest and most flexible probe, not only in the mouth, but even with the teeth extracted and in the hand.

I have shown so many of these in clinics that their existence has come to be a matter of course, exemplified not only by excessive tortuosities, but even more markedly by the "bayonet" and "adjunctive" roots, the canals of which latter frequently open at right angles into the canals from which they diverge, thus rendering it not only impossible to follow, but equally impossible to know of their existence until after the extraction of the teeth.

And yet it is with these, as with the others, that we sometimes have to deal, and it is not infrequent that we find such complications associated with the very teeth which we most desire to save.

For the filling of the most difficult and most unsatisfactory of these canals I should suggest the most searching, soothing and *permanently antiseptic* medicaments, such as oil of cinnamon or cloves, eugenol and campho-phenique, and for probational purposes such medicaments as electrosone, borolyptol and solutions (two to four per cent.) of formalin, not as curative, but as prophylactic treatment.

For the filling of the less difficult and yet not satisfactorily prepared canals, I would again suggest pure medicinal fillings in order that the greatest amount possible of some material that may *do some good* shall be introduced, but I would also suggest that a longer continuance of benefit may be assured by greater consistency of material, and for this purpose I employ "pastes" of varying degrees of inspissation, composed of acetate of morphia, sulphite of lime, antipyrine, or fluo-silicate of soda softened by oil of cloves or cinnamon or campho phenique, and *probationally*, balsamo del deserto, or even salol.

For the filling of the accessible, large and easily entered canals, it is found better that a vehicle for the introduction of the medicament should be used, for the double reason that a sufficiency of antiseptic can thus be placed, and that a better preventive to ingress of fluid can thus be provided.

It was early noted that the "pinhead pellets" of cotton, which almost always preceded the excellent gold canal fillings of the earlier operators, if removed for relief to subsequent peridental trouble, came forth permeated with the odor of putrefaction from remaining portion of pulps, as the extirpation had been more or less thorough, but in either case its *structural integrity seemed* perfect.

I argued that if this was maintained in pellet form, it might also be in form of taper-twist, and, therefore, used *natural cotton* (not absorbent) as the vehicle for medication, and the taper-twists were gradually increased in size until each *almost completely* filled its canal.

In normally formed roots this material was found, by experiment, to subserve nicely, the purpose of excluding moisture, and in cases of large foramina or unformed roots a portion of base-plate, and in later years, temporary stopping, was incorporated with the small end of the taper-twist, and being warmed, was pressed into place; the cotton was medicated and packed firmly as usual.

Year after year has increasingly demonstrated the wisdom and utility of this practice, as hundreds of teeth thus treated grew into thousands, and the thousands into more than ten thousand; and of these, treated and re-treated quickly, easily and comfortably during the past thirty years, I do not know of *fifty* which have been lost.

I was up at Medford visiting a young friend of mine who has now been married about twenty-six years. I was speaking with him of a tooth which I had treated for him before he was married. Referring to this lower molar which came to me in a badly diseased condition, I said to him: "Joshua, how about that tooth which I treated for you some twenty-six years ago, I suppose it has all gone." He said: "The top has, doctor, but the roots are there."

What a commentary that was upon the treatment of the tooth. We all recognize that it is the roots which give trouble, and his reply was: "The top is all gone, but the roots are there."

Into the mouth of each canal a tiny pellet of cotton is introduced, which is called a "guard ball," as it more surely than in any other way guards against sudden or unintentional withdrawal of a canal filling.

Upon these guard balls a filling of temporary stopping is inserted, covered in turn with whatever resistant material may be indicated.

It is now twenty-four years since, desiring acceptable, corroborative testimony regarding the maintenance of integrity of the *vehicle* which I was using, I submitted removed cottons of five, six, seven and eleven years' service to Professor Joseph G. Richardson, M. D., then microscopist at the University of Pennsylvania. The following was his report:

PHILADELPHIA, May 16, 1872.

*Professor J. Foster Flagg, D. D. S.*:

DEAR SIR:—On examining the portions of cotton which you handed me in your office, and which you informed me had been imbedded beneath plugs, in the fangs of teeth, for five, six, seven and eleven years, respectively, I found that single filaments unraveled from the middle and each extremity of the last of these fragments (entombed eleven years ago) when investigated both dry and wet, under powers of 220, 440, 1200 and 2400 diameters, displayed clearly the ordinary structure of cotton fibre and *exhibited no evidence of disintegrative change.*

The other pieces, similarly inspected, were likewise apparently unaltered.

Very respectfully yours, etc.,

(Signed) JOSEPH G. RICHARDSON, M. D.

Again, desiring further testimony as to *continued* integrity, I submitted to professor Ernest Laplace, M. D., microscopist of the Medico-Chirurgical College, during the winter of 1893-94, six specimens for microscopic examination.

Of these No 1 was a new, unused "taper-twist."

No. 2. A cotton canal filling of over ten years' service.

No. 3. Cotton canal filling of twenty-two years' service—left upper molar.

No. 3. Cotton canal filling of seventeen years' service —right upper bicuspid.

No. 5. Cotton canal dressing of one week.

No. 6. Cotton canal filling of twenty-three years' service—left lower molar.

The results of his examinations were given to me by him as follows:

Results of Professor Laplace's examinations:

No. 1. Twisted fibres for filling material—new; contains little foreign substance, thicker at small end. This foreign material probably dust.

No. 2. Piece of cotton from a canal—ten years old; no apparent disintegration of cotton fibre; large amount of amorphous foreign material adherent to fibre; more of this material toward tip than further up.

No. 3. Piece of cotton dressing from left upper molar —twenty-two years; no apparent disintegration of fibre; foreign material more gathered in lumps; not disseminated like former.

No. 4. From right upper cuspid—seventeen years; about same amount of foreign material as No. 4, but not so massed or lumpy; no apparent disintegration of cotton fibre.

No. 5. Cotton dressing for treating—one week old; fibres, both singly and collectively, are covered with foreign material; foreign material around fibres joining them together; fibres good.

No. 6. From left lower molar—twenty-three years; most of fibres free from foreign material; here and there masses of this foreign material.

Professor Laplace offered to have made two microphotographs from No. 1 and from No. 6, and said that no one could tell the difference. I thanked him and told him it was *sufficient that he had said so.*

I have felt it my mission to bring all this before you, because there are those who tell patients that their teeth give them trouble *because they were filled with cotton. This is not true.* And I am glad that thousands of patients know that it is not.

I never told patients that their teeth gave trouble *because* they were filled with gold. But I have told hundreds of patients that the long, tedious, painful road to relief *was because* of these gold canal fillings, and that was true.

And I have told them that I would so fill them that, if trouble should come again, relief would be quickly, and easily, and comfortably given, *and they have found it so.*

I have worked sometimes unsuccessfully in efforts to remove badly misused amalgam and wretched chloro-percha, "thoroughly" pumped in. And I have regretted sincerely, for the patients' sakes, that such work had been done.

I have in my collection roots of teeth which have beautiful fillings of gold in their canals, introduced as "better practice," after removal of my canal cotton for relief. And when trouble again came, *caused by the gold*, so "excellent" was the work that relief could not be given. The crowns were broken off in attempts to extract, and the roots were brought to me by the luckless patients for ether, chloroform, and removal.

It is strange that I wear these scalps at my girdle as trophies gathered on the war path? Were I more than human I might not do so, but I am not.

"Cotton canal filling" has been to me and mine a blessing beyond computation; having given it to a patient and having explained its "why and wherefore," I feel that I have clearly, intelligently and "thoroughly" done my duty, in that I have done well, as shown by clinic experience, not only for the present, but that I have provided, in the best possible degree, for years of comfort, and, vastly more than this, for *prompt relief, bestowed most gently*, in the event of future trouble.—*Items of Interest.*

